# Biomimetic ZIF8 Nanosystem With Tumor Hypoxia Relief Ability to Enhance Chemo-Photothermal Synergistic Therapy

**DOI:** 10.3389/fphar.2022.850534

**Published:** 2022-03-24

**Authors:** Ziming Zhao, Zhaorong Liu, Yabing Hua, Yuanjie Pan, Ge Yi, Shengyue Wu, Cong He, Yanzhuo Zhang, Yihua Yang

**Affiliations:** ^1^ Jiangsu Key Laboratory of New Drug Research and Clinical Pharmacy, Xuzhou Medical University, Xuzhou, China; ^2^ Department of Pharmaceutics, School of Pharmacy, Xuzhou Medical University, Xuzhou, China

**Keywords:** biomimetic, ZIF8, photothermal therapy, chemotherapy, hypoxia relief

## Abstract

Tumor hypoxic microenvironment can reduce the therapeutic effects of chemotherapy, radiotherapy, photodynamic therapy, immunotherapy, etc. It is also a potential source of tumor recurrence and metastasis. A biomimetic nanosystem based on zeolitic imidazolate framework 8 (ZIF8), which had multifunctions of hypoxia relief, chemotherapy, and photothermal therapy, was established to improve tumor hypoxic microenvironment and overcome the corresponding therapeutic resistance. ZIF8 enveloped with DOX and CuS nanoparticles (DC@ZIF8) was synthesized by a sedimentation method. Red blood cell membrane and catalase (CAT) were coated onto DC@ZIF8 and biomimetic nanosystem (DC@ZIF8-MEM_C_) was formed. The designed DC@ZIF8-MEM_C_ had a shape of polyhedron with an average particle size around 254 nm. The loading content of DOX, CAT, and CuS was 4.9%, 6.2%, and 2.5%, separately. The release of DOX from DC@ZIF8-MEM_C_ was pH dependent and significantly faster at pH 5 due to the degradation of ZIF8. DC@ZIF8-MEM_C_ exhibited outstanding photothermal conversion properties and excellent antitumor effect *in vitro* and *in vivo.* Moreover, the hypoxia relief by CAT was proved to have good sensitization effect on chemo-photothermal combined therapy. DC@ZIF8-MEM_C_ is a prospective nanosystem, which can realize great chemo-photothermal synergistic antitumor effect under the sensitization of CAT. The biomimetic multifunctional nanoplatform provides a potential strategy of chemo-photothermal synergistic antitumor effect under the sensitization of CAT.

## Introduction

Cancer has become one of the major diseases threatening human health and life (Li D. et al., 2021). The conventional clinical methods, such as chemotherapy (CT), surgery, radiation therapy, have made remarkable achievement in the treatment of cancer. However, therapeutic outcomes are unsatisfactory, and patient compliance is poor, resulting from far-reaching side effects (Li et al., 2019). Researchers have devoted to develop new approaches, such as photothermal therapy (PTT), photodynamic therapy (PDT), chemodynamic therapy (CDT), immunotherapy, gene therapy, and molecular targeted therapy.

As one of the rising “green” cancer treatment methods, PTT based on photothermal agents converts energy from near infrared (NIR) light into heat, leading to overheating of the surrounding environment and inducing cancer cell death (Chen et al., 2019; Jiang et al., 2019). Photothermal agents, such as gold nanorods (He et al., 2021), carbon nanorods (Zhao et al., 2021), palladium nanorods (Tang et al., 2014), semiconducting polymer nanoparticles (Zhen et al., 2018), NIR dyes (Yue et al., 2013; Zhao et al., 2014), polydopamine (PDA) (Ding et al., 2016), and copper sulfide (CuS) (Jang et al., 2018; Shi et al., 2018; Li J. et al., 2021) have been reported as having good photothermal effect. However, PTT may lead to uneven heat distribution and subsequent sublethal thermal dose in the treatment area (Jiang et al., 2019; Zhang L. et al., 2019). Moreover, utilizing PTT alone to treat tumor is suboptimal and unreliable. Generally, co-delivery of chemotherapeutic drug and photothermal agent simultaneously exerts two benefits to improve antitumor efficacy. As is well known, a combination therapy of CT and PTT based on nanomaterials exhibits significant advantages over monotherapy for anticancer treatment and usually generates synergistic therapeutic efficacies with minimal side effects (Liu et al., 2011; Li et al., 2018; Li et al., 2019).

CT based on nanocarrier to deliver a chemical drug is effective. As a new kind of porous material, ZIF8 is built from zinc ions and 2-methylimidazolate, which are components of physiological systems (Zhuang et al., 2014). It possesses unique merits including significantly high microporosity, excellent structural regularity, adjustable surface functionality, and intrinsic pH-induced biodegradability (Sun et al., 2012; Liu H et al., 2021). In addition, it is prone to decompose under acidic conditions (pH 5.0–6.0), which is helpful to the pH-controlled delivery and release of the loaded payloads (Park et al., 2006; Lian et al., 2017; Gao et al., 2019). A theranostic nanoplatform based on ZIF8 encapsulated Pd nanosheets and DOX (DOX/Pd@ZIF8@PDA) showed photoacoustic (PA) imaging-guided synergetic photo-chemo cancer therapy (Zhu et al., 2019). A drug delivery nanoplatform based on ZIFs (PDA-PCM@ZIF8/DOX) exhibited drug release as high as 78% under the dual stimulus of NIR and acid environment (Wu et al., 2018).

Moreover, hypoxia is a unique feature of tumor microenvironment due to rapid cell proliferation and abnormal vascular structure (Vaupel and Mayer, 2016; Graham and Unger, 2018). Numerous studies have demonstrated that tumoral hypoxia can activate the overexpression of P-glycoproteins (P-gp), which markedly reduced the effect of CT (Samanta et al., 2014.). Hypoxia also induces tumor gene mutations and subsequent proteomic changes, ultimately impeding the clinical therapeutic effect of cancer therapy (Jing et al., 2019). Additionally, cancerous cells produced excessive amounts of hydrogen peroxide (H_2_O_2_) in tumor regions, resulting in oxide stress (Szatrowski and Nathan, 1991; Song et al., 2016). Using H_2_O_2_-responsive enzyme is an intriguing strategy to overcome tumor hypoxia (Szatrowski and Nathan, 1991; Chen et al., 2014; Zhang et al., 2017; Zou et al., 2018; Yin et al., 2020). CAT, an enzyme, is capable of catalyzing H_2_O_2_ to oxygen (O_2_) efficiently, leading to tumor hypoxia relief (Liu et al., 2019; Yin et al., 2020).

**FIGURE 1:**
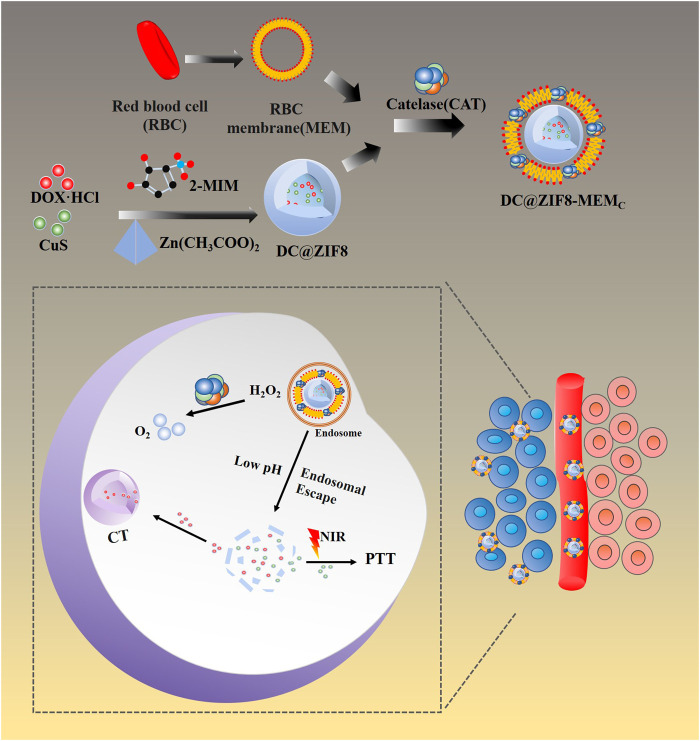
Schematic illustration for the formation of DC@ZIF8-MEM_C_ nanoplatform.

Herein, we developed ZIF8 NPs to load CuS NPs and DOX·HCl, then camouflaged with bionic surface material, red cell membrane (MEM) combined with CAT, to construct a biomimetic nanosystem with multifunctions of chemotherapy, hyperthermia treatment, and hypoxia relief (Figure 1).

## Materials

### Reagents

Copper (II) chloride dihydrate (CuCl_2_·2H_2_O, analytical grade), sodium citrate dihydrate (Na_3_C_6_H_5_O_7_·2H_2_O, analytical grade), sodium sulfide nonahydrate (Na_2_S·9H_2_O, analytical grade), and polyvinylpyrrolidone K30 (PVP K30, analytical grade) were obtained from Sinopharm Chemical Reagent Co., Ltd. (Shanghai, China). Zinc acetate dihydrate [Zn (CH_3_CO_2_) · 2H_2_O, 98%], 2-methylimidazole (98%), catalase (CAT) were purchased from Aladdin Chemistry Co., Ltd. (Shanghai, China). Tris (4,7-diphenyl-1,10-phenanthroline)-ruthenium (II) dichloride [Ru (dpp)_3_Cl_2_, 98%] was obtained from Rhawn Reagent Co., Ltd. (Shanghai, China). Doxorubicin hydrochloride (DOX·HCl, 98%) was supplied from Aladdin Chemistry Co., Ltd. (Shanghai, China). BCA Protein Assay Kit, 3-[4,5-dimethylthiazol-2-yl]-2,5-diphenyltetrazoliumbromide (MTT, 98%) and hoechst 33258 were obtained from Beyotime Biotechnology Co., Ltd. (Shanghai, China). Cell Viability/Cytotoxicity Detec (Calcein AM/PI) was obtained from Yuanye Bio-Technology Co., Ltd (Shanghai, China).

### Cells

Mouse breast cancer cells (4T1) and leukemia cells in mouse macrophage (RAW264.7) were obtained from the Chinese Academy of Sciences (Shanghai, China). The 4T1 cells were incubated with DMEM/f12 medium containing 10% FBS, 1% streptomycin (50 U·mL^−1^), and penicillin (50 U·mL^−1^) in a 5% CO_2_ atmosphere at 37°C. The RAW264.7 cells were cultured in DMEM medium supplemented with 10% FBS, 1% streptomycin (50 U·mL^−1^), and penicillin (50 U·mL^−1^) in a 5% CO_2_ atmosphere at 37°C.

### Animals

BALB/c female mice (20 ± 2 g) were purchased from the Laboratory Animal Center of Xuzhou Medical University (Xuzhou, China), and maintained in a sterile environment, and allowed free access to food and water, and all experiment procedures were approved by the Experimental Animal Ethics Committee of Xuzhou Medical University and carried out by the guidelines of the National Act on the Use of Experimental Animals (People’s Republic of China).

## Methods

### Preparation of DC@ZIF8-MEM_C_

#### Preparation of CuS NPs

CuCl_2_·2H_2_O (0.4 mM) and Na_3_C_6_H_5_O_7_·2H_2_O (0.27 mM) were dissolved in 180 ml of deionized water, and then 20 ml of Na_2_S·9H_2_O (0.4 mM) aqueous solution was added to the mixed solution. After stirring for 5 min at room temperature, the mixed solution was stirred for 15 min at 90°C, until dark green was obtained. The synthesized product was centrifuged and then washed with deionized water three times yielding CuS NPs.

#### Preparation of DC@ZIF8

2-Methylimidazole (460 mg) and PVP K30 (25 mg) were dissolved in 10 ml of CuS aqueous solution (2 mM), 1 ml of zinc acetate methanol solution (0.56 mM) was added and stirred for 10 min, centrifuged, dried under vacuum to obtain CuS NPs-loaded ZIF8 NPs (C@ZIF8). DOX·HCl (1 mg) was mixed with 12 mg C@ZIF8, dissolved in distilled water, and stirred for 10 min, centrifuged, and washed to collect DC@ZIF8.

#### Extraction of Red Blood Cell Membrane

Red blood cell (RBC) membranes were obtained according to a reported method with modification (Piao et al., 2014). Fresh whole blood from male ICR mice (20–22 g) was collected with heparinized tubes, followed by centrifugation for 10 min with 4,000 rpm at 4°C to remove the plasma and the leukocytes. The collected red blood cells (RBCs) were washed with 1× PBS, suspended with 0.25× PBS for 30 min at 4°C, and then centrifuged at 10,000 rpm for 10 min. The resulting light-pink pellet was purified with 1× PBS, which yielded RBC membrane (Piao et al., 2014).

### Preparation of DC@ZIF8-MEM_C_

The above prepared DC@ZIF8 and CAT were dispersed in 10 ml of RBC membrane solution and then ultrasonicated with ice bath for 6 min. The precipitation of DC@ZIF8-MEM_C_ was collected by washing and centrifugation, and placed at 4°C for later use. DC@ZIF8-MEM was prepared by the same method above except for the absence of CAT.

### Characterization

#### Morphology, Size, and Zeta Potential

The particle size and zeta potential were determined by dynamic light scattering (DLS) method using a NicompTM 380 ZLS zeta potential/size analyzer (Particle Sizing Systems, Santa Banta, CA, USA) at 25°C. The morphologies of the NPs were observed using transmission electron microscopy (TEM) (FEI Tecnai G2 Spirit Twin, Holland).

### Load Efficiency

The loading content (LC) was calculated using the following formula: LC = weight of loaded drug/weight of NPs*100%. DOX·HCl was quantitatively analyzed using fluorescence spectrum (excitation wavelength = 488 nm, emission wavelength = 560 nm). The quantification of CAT was determined by ultraviolet spectrophotometer based on ammonium molybdate colorimetric method. The NPs were digested by nitric acid, and Cu^2+^ content was detected by an inductively coupled plasma spectrometer (PQ9000, German), and then the LC of CuS was analyzed.

### Spectra Analysis

Infrared spectra and ultraviolet (UV)-visible (UV-vis) spectra were obtained by using a Fourier transform infrared spectrometer (FTIR, BRUKER) and an Evolution 220 UV-Visible Spectrophotometer (Mapada Instruments, Shanghai), respectively.

#### Drug release *in vitro*

The release study was assessed by the dialysis method. The release media was PBS solutions with different pH values (5.0, 6.5, and 7.4). Briefly, 5 ml of DOX·HCl-loaded NPs was placed in a dialysis bag (MWCO 8,000–14,000) and dialyzed against 45 ml of buffer medium under mechanical shaking (100 rpm) at 37°C. At predesigned time, 5 ml of release medium was withdrawn and replenished with an equal volume of fresh medium. The released DOX·HCl was detected by Fluorescence spectrum.

### pH Sensitivity

DC@ZIF8-MEM_C_ was incubated with 10 mM PBS (pH 7.4 or pH 5.0) at 37°C for 8 h, and the morphology or size was detected.

### Stability

DC@ZIF8-MEM_C_ was suspended in PBS (pH 7.4, 10 mM) or 10% fetal bovine serum (FBS), and measurements were performed in triplicate at different intervals.

#### Photothermal Activity *in vitro*

The photothermal conversion capability of DC@ZIF8-MEM_C_ was investigated. Various concentrations of DC@ZIF8-MEM_C_ (0, 60, 125, 250 μg·mL^−1^) were dispersed in 1 ml of deionized water and irradiated by 808 nm NIR laser at 2 W·cm^−2^ for 5 min. The temperature of each sample was monitored by a FLIR imaging instrument.

DC@ZIF8-MEM_C_ (250 μg·mL^−1)^ was dispersed in 1 ml of deionized water and irradiated by 808 nm NIR laser at different power densities (0.5, 1.0, 1.5, 2 W·cm^−2^) for 5 min. The temperature of each sample was monitored by a FLIR imaging instrument.

To evaluate the photothermal stability of NPs, 1 ml of DC@ZIF8-MEM_C_ was irradiated by 808 nm laser (2 W·cm^−2^) for four laser ON/OFF cycles. The photothermal conversion efficiency (η) of DC@ZIF8-MEM_C_ was calculated as follows according to a previously reported method (Zhang X. et al., 2019):

$$\text{η}=\frac{hS\left(T_{max}-T_{surr}\right)-Q_{dis}}{\text{I}\left(1-10^{-\text{A}808}\right)}\times 100\%$$

where h represents heat-transfer coefficient, S represents the irradiated area, T_max_ represents the equilibrium temperature, T_surr_ is ambient temperature of the surrounding, Q_dis_ is the heat associated with the light absorbance of the solvent, I is the power density of the laser (2 W·cm^−2^), and A_808_ is the absorption value of the material at 808 nm (Zhang X. et al., 2019). Photothermal conversion of DC@ZIF8 was performed the same above.

### O_2_ Generation *in vitro*

The fluorescence of O_2_-sensitive fluorescent probe Tris (4,7-diphenyl-1,10-phenanthroline) ruthenium (II) dichloride [Ru (DPP)_3_Cl_2_] will be quenched to a certain extent when O_2_ concentration is sufficient. H_2_O_2_ solution (10 mM), Ru (DPP)_3_Cl_2_ solution (1 mM), and DC@ZIF8-MEM_C_ (125 μg·mL^−1^) were mixed and sealed. The emission spectra were scanned at a predetermined time (0, 10, 20, 30, 45, 60 min).

Additionally, the portable dissolved oxygen meter was used to measure the *in situ* O_2_ generation. Free CAT, DC@ZIF8-MEM_C_, DC@ZIF8-MEM, and PBS were mixed with H_2_O_2_ solution (10 mM), and the O_2_ production was dynamically detected by the portable dissolved oxygen meter (JPBJ-609L, INESA Scientific Instrument Co., Ltd., China) every 30 s for 600 s.

### Catalase activity

Catalase activity was evaluated by the Goth method (Goth, 1991). Free CAT, DC@ZIF8-MEM_C_ [(CAT) = 2.5 μg·mL^−1^] and DC@ZIF8-MEM were incubated with H_2_O_2_ solution (50 mM) at 37°C, then ammonium molybdate was added to terminate the reaction, and the absorbance was determined at 400 nm according to a standard curve of H_2_O_2._ CAT was reliable to degrade by the protease K under complicated physiological conditions (Phua et al., 2019). Free CAT and DC@ZIF8-MEM_C_ was incubated with protease K (100 μg·mL^−1^) at 37°C, and then the absorbance was determined according to the method above.

### Cell Experiments

#### Cellular Uptake

4T1 cells were seeded at a density of 2 × 10^4^ cells/well in 48-well plates and treated with samples (DOX·HCl, DC@ZIF8, DC@ZIF8-MEM_C_) at an equivalent DOX·HCl concentration of 1 μg·mL^−1^, respectively. After incubation for 4 h, the cells were washed by PBS, and then the cell nuclei were stained with Hoechst 33258 for 10 min. Afterward, the cells were observed under an inverted fluorescence microscopy.

The cellular uptake was quantified using a microplate reader following the procedure (Win and Feng, 2005). 4T1 cells were seeded in 96-well plates at a density of 1 × 10^4^ cells/well. After cell attachment, fresh media containing different formulations [(DOX·HCl) = 1 μg·mL^−1^] were added and incubated for 4 h. After washing with PBS, the cells were treated with 0.5% of TritonX-100 solution for 30 min. Fluorescence intensity of cell lysates (E_x_ = 480 nm, E_m_ = 595 nm) was determined by a fluorescence microplate. Uptake efficiency (%) = W_sample_/W_total_ *100%

#### Cytotoxicity *in vitro*

The *in vitro* cytotoxicity was evaluated by using MTT assay. 4T1 cells pre-seeded at a density of 1 × 10^4^ cells/well in 96-well plates were incubated with different samples at gradient concentrations for 24 h. For NIR irradiation treatment, cells were irradiated by 808 nm for 3 min (2 W·cm^−2^) after 6 h of incubation and then incubated for further 18 h. Then 20 μl of MTT was added and incubated for another 4 h. Subsequently, all of the solutions were replaced with 150 µl dimethyl sulfoxide (DMSO). The absorbance was determined by using a microplate reader at 490 nm. The untreated cells in medium were regarded as control. The cell viabilities (%) were calculated according to the reported literature (Zou et al., 2018).

4T1 cells seeded at a density of 1 × 10^4^ cells/well in 96-well plates and incubated at different samples with hypoxic incubator (1% O_2_) for 24 h. Then 10 µM H_2_O_2_ was added, and the cell viabilities were calculated the same as above.

Calcein AM/PI double staining kit was performed to evaluate live/dead cells (Liu et al., 2019). The 4T1 cells were seeded at a density of 1 × 10^4^ cells/well in 96-well plates and cultured overnight. The media were replaced with DOX·HCl, DC@ZIF8-MEM_C_ [(DOX·HCl)] = 3.5 μg·mL^−1^], and incubated for 24 h. For the laser group, the cells were irradiated by 808 nm NIR (2 W·cm^−2^) for 3 min after 8 h of incubation, and then continued for 16 h of incubation. Calcein-AM (2 μM) and PI (4.5 μM) were added and incubated for 30 min. The cells were washed with PBS and observed under an inverted fluorescence microscope.

### Intracellular Location

4T1 cells were inoculated and incubated overnight at a density of 1 × 10^5^ cells/dish in laser confocal Petri dishes. Fresh medium containing DC@ZIF8-MEM_C_ [(DOX·HCl) = 1 μg·mL^−1^] was added and incubated for 0.5, 2, 6 h, respectively. The samples were washed with PBS and then stained with Lysotracker for 20 min and Heochst-33258 for 10 min. Intracellular location was observed by confocal laser microscopy (CLMS) and analyzed by ImageJ software (Zhu et al., 2018).

### Immune Evasion

RAW 264.7 cells were selected as a macrophage model to demonstrate that the composite vector coated by blood cells can reduce the probability of being phagocytosed by macrophages. RAW 264.7 cells were seeded at density of 1 × 10^4^ cells/well in 96-well plates and incubated overnight. Different samples (DOX·HCl, DC@ZIF8, and DC@ZIF8-MEM_C_, with DOX·HCl concentration 1 μg·mL^−1^) were added, and incubated for 4 h. The medium was discarded, and Heochst-33258 was used as a stain for 10 min. Cell uptake was observed by inverted fluorescence microscopy. The quantitative uptake was investigated similarly as described above.

### Intracellular Oxygen Determination

4T1 cells were seeded at a density of 4 × 10^4^ cells/well into 24-well plates, and 10 µM H_2_O_2_ was added for 2 h with a hypoxic incubator. Different samples were added and incubated for 4 h. Ru (DPP)_3_Cl_2_ (0.5 µM) was added for 1 h and observed by an inverted fluorescence microscopy.

### Tumor Inhibition Effect *in vivo*

The animal model was built by subcutaneous injection with 100 µl of 4T1 cells (1 × 10^6^ cells) in the right hind limb of female BALB/c mice. The tumor-bearing mice were randomly divided into seven groups (each group had five mice with similar tumor volume about 200 ± 20 mm^3^): 1) PBS group, 2) DOX·HCl group, 3) C@ZIF8-MEM group; 4) D@ZIF8-MEM group; 5) DC@ZIF8-MEM + NIR group; 6) DC@ZIF8-MEM_C_ group; 7) DC@ZIF8-MEM_C_ + NIR group. The mice were treated with PBS (group 1), NPs (groups 2 to 7, with a dosage of DOX·HCl 5 mg·kg^−1^) *via* tail vein injection at day 0, 3, 6, and 9 for an overall of four times. For the NIR groups, the mice were treated with NIR 808 nm irradiation (2 W·cm^−2^) at the tumor site for 5 min after 8 h post-injection. Tumor volume was calculated by the formula (a × b^2^)/2, where a and b are the long and short diameters of the tumor, respectively, which were measured every other day by a vernier caliper (Wu et al., 2018).

On day 14, the mice were sacrificed, and major organs, including the heart, liver, spleen, lungs, kidneys, and tumor were collected for pathological evaluation.

### Photothermal Effect *in vivo*

The tumor-bearing mice were tail vein injected with PBS, DC@ZIF8, DC@ZIF8-MEM, and DC@ZIF8-MEM_C_. After an 8 h-injection, the mice were anesthetized with 40 μl of 10% chloral hydrate by intraperitoneal injection. The tumor site of the mice was irradiated by 808 nm of NIR (2 W·cm^−2^) for 5 min. Simultaneously, the photothermal imaging of the tumor site was recorded by the near-infrared imager. The temperature change in the tumor site with time was observed, and the temperature curve was plotted.

### Hemolysis Assay

The hemocompatibility was evaluated by hemolysis assay (Yang et al., 2019). First, fresh blood extracted from rabbit heart was centrifuged at 3,000 rpm for 15 min and purified with normal saline (NS) to obtain erythrocytes. DC@ZIF8-MEM_C_ with various concentrations were mixed with the 2% erythrocytes (v/v) and incubated for 3 h. Afterward, the mixtures were centrifuged at 3,500 rpm for 15 min, and the absorbance (A) of the sample supernatant at 540 nm was measured by UV-vis spectrophotometer. PBS (0.01 M, pH 7.4) and deionized water were regarded as the negative and positive controls, respectively. The hemolysis ratio (HR) was calculated as follows: HR (%) = (A_sample_ − A_negative control_)/(A_positive control_ − A_negative control)_ * 100%.

### Statistical Analysis

The data were expressed as the mean ± standard deviation (SD). Statistical analysis was performed using a two-tailed Student’s t-test and analysis of variance (ANOVA) with the software SPSS 23.0. All analyses were shown compared with the control, and the significance of the difference was indicated as *p < 0.05, **p < 0.01, or ***p < 0.001.

## Results and discussion

### Synthesis and Characterization

**FIGURE 2:**
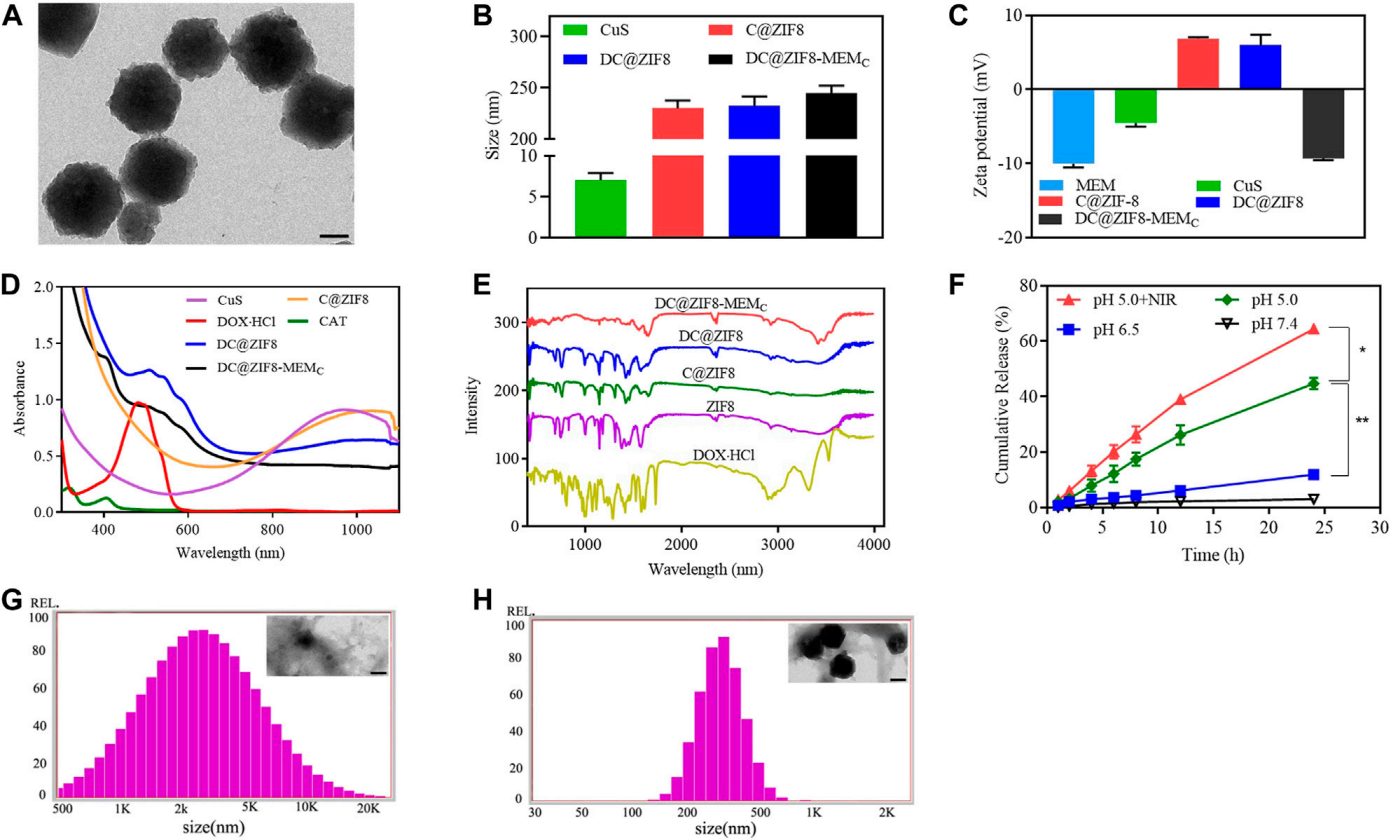
Representative transmission electron microscopy (TEM) image of DC@ZIF8-MEM_C_ (scale bar = 100 nm) **(A)**. Size of preparations **(B)**. Zeta potential of preparations **(C)**. Ultraviolet (UV)-visible (UV-Vis) analysis **(D)**. Fourier transform infrared (FTIR) analysis **(E)**. DOX·HCl release profiles from DC@ZIF8-MEM_C_ under PBS at pH values of 5.0, 6.5, and 7.4 **(F)**. Size and TEM image (inserted) of DC@ZIF8-MEM_C_ with PBS (pH 5.0) for 8 h; scale bar = 100 nm **(G)**. Size and TEM image (inserted) of DC@ZIF8-MEM_C_ with PBS (pH 7.4) for 8 h; scale bar = 100 nm **(H)**.

CuS NPs appeared dark green (Supplementary Figure S1). Both DC@ZIF8 and DC@ZIF8-MEM_C_ showed purplish red due to the loaded DOX·HCl (Supplementary Figure S1). CuS NPs were spherical, while DC@ZIF8 was polyhedral due to the frame of ZIF8 by TEM (Supplementary Figure S2). After coating with MEM-embedded CAT, DC@ZIF8-MEM_C_ maintained a polyhedral structure by TEM (Figure 2A). The size of CuS NPs was only 7 nm, and C@ZIF8 increased to 230 nm due to the framework of ZIF8 (Figure 2B). After drug loading, the size of DC@ZIF8 was 232 nm, and the size of DC@ZIF8-MEM_C_ increased to 254 nm for the coating of cell membrane (Figure 2B). Both C@ZIF8 and DC@ZIF8 were positive zeta potential (Figure 2C). However, the zeta potential of DC@ZIF8-MEM_C_ reversed to be negative (−9.3 mV), demonstrating a successful membrane coating (Figure 2C). DOX·HCl was quantitatively analyzed by fluorescence spectrum (Supplementary Figure S3). The CAT was quantitatively analyzed by ultraviolet spectrophotometer (Supplementary Figure S4). The LC of DOX, CAT, and CuS was 4.9%, 6.2%, and 2.5%, separately. Moreover, DC@ZIF8-MEM_C_ exhibit good colloidal stability in both PBS and 10% FBS, as indicated by the negligible increase in hydrodynamic diameter (Supplementary Figure S5).

UV-Vis analysis showed that DC@ZIF8-MEM_C_, DC@ZIF8, and CuS NPs all had a very wide absorption band in the near infrared region from 800 to 1,100 nm. Therefore, a near-infrared laser of 808 nm can be used as a heat source for photothermal effect. DC@ZIF8-MEM_C_ had characteristic absorbance peaks from DOX (480 nm) and CAT (400 nm), confirming the successful loading of these two materials (Figure 2D).

By recording Fourier transform infrared (FTIR) spectra, the prepared DC@ZIF8-MEM_C_ was further characterized. As depicted in Figure 2E, DC@ZIF8-MEM_C_ exhibited a characteristic peak of 1,700 cm^−1^, which was the C=O stretching vibration of sodium citrate in CuS NP_S_, and a –CH stretching vibration peak at 2,800 cm^−1^, which was consistent with the characteristic peak of DOX·HCl. DC@ZIF8-MEM_C_ had a characteristic peak of 3,500–3,700 cm^−1^, which was abundant –OH peaks on the surface of CAT and heterozygous membrane. The results of FTIR spectrum analysis and UV analysis were consistent, which showed that DC@ZIF8-MEM_C_ was successfully constructed.

Drug release *in vitro* showed pH-dependent manners (Figure 2F). The cumulative release of DOX·HCl in pH 7.4, pH 6.5, and pH 5.0 media at 24 h was 3.1% 11.9%, and 44.7%, respectively. There was little drug release in pH 7.4, indicating that the developed NPs were stable under physiological conditions. Compared with pH 5.0 group, release from pH 5.0 + NIR group was higher, indicating that NIR accelerated the drug release. Interestingly, pH sensitivity of DC@ZIF8-MEM_C_ was evident from Figures 2G, H. When DC@ZIF8-MEM_C_ was in a neutral condition (pH 7.4 PBS), the morphology and size changed little for 8 h (Figure 2H). It degraded under acidic conditions (pH 5.0), and the size increased to 2,000 nm, 10-fold bigger than that of PBS (pH 7.4) (Figure 2G). Under acidic conditions, the release amount of the drug was significantly increased, which may be related to the degradation of the material at lower pH value (Wu et al., 2018). The imidazole groups from ZIF8 were protonated in an acidic environment, and the unstable coordination bonds between zinc ions and imidazole groups became unstable, leading to the disintegration of the skeleton and acceleration of the drug release.

### Photothermal Properties

**FIGURE 3:**
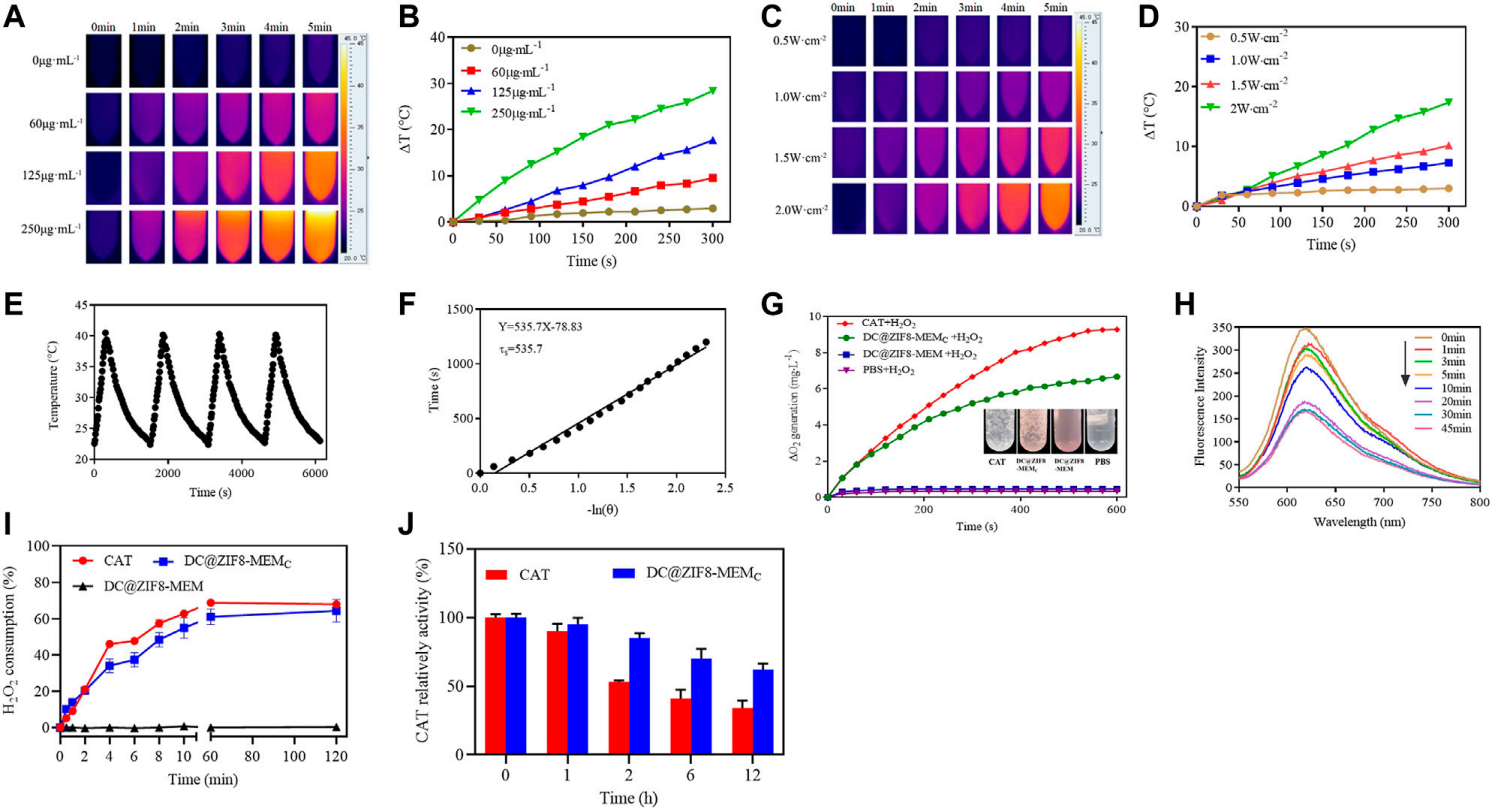
Photothermal images of DC@ZIF8-MEM_C_ with various concentrations by FLIR **(A)**. Temperature curves by changing concentration of DC@ZIF8-MEM_C_ with 808 nm near infrared (NIR) at 2 W·cm^−2^ for 5 min **(B)**. Photothermal images of DC@ZIF8-MEM_C_ with various laser power densities by FLIR **(C)**. Temperature curves of DC@ZIF8-MEM_C_ by changing the laser power density **(D)**. Temperature variations of DC@ZIF8-MEM_C_ by repeating on/off (2 W cm^−2^) **(E)**. Temperature fitting curve of DC@ZIF8-MEM_C_ **(F)**. The O_2_-generating behavior of DC@ZIF8-MEM_C_, DC@ZIF8-MEM, PBS, and free CAT solution in the presence of H_2_O_2_. Inset is the corresponding image of these four solutions **(G)**. Fluorescence absorption curves of ruthenium (II) dichloride [Ru (DPP)_3_Cl_2_] incubation with DC@ZIF8-MEM_C_ **(H)**. The H_2_O_2_ consumption kinetics of free catalase (CAT), DC@ZIF8-MEM_C_, and DC@ZIF8-MEM at 37°C **(I)**. The relative enzymatic activity of free CAT and DC@ZIF8-MEM_C_ after treating with protease K for different incubation times **(J)**.

As shown in Figures 3A, B, the temperature rise followed a concentration-dependent manner. The temperature of the water increased by only 2.1°C. At concentrations of 60, 125, 250 μg·mL^−1^, DC@ZIF8-MEM_C_ increased by 9.5°C, 17.7°C, and 28.4°C, respectively (Figures 3A, B). Furthermore, the temperature increased with power. The temperature increased by 17.4°C when the concentration was 125 μg·mL^−1^, and the power density of the 808 nm laser was 2.0 W·cm^−2^ (Figures 3C, D).

Moreover, by measuring the rising/falling temperature, it was found that the photothermal conversion was stable. The photothermal conversion efficiency was calculated to be 51.5%, consistent with a report (Shi et al., 2018), indicating the excellent photothermal performance of DC@ZIF8-MEM_C_ (Figures 3E, F). Intriguingly, the photothermal conversion efficiency of DC@ZIF8 was 54%, similar to that of DC@ZIF8-MEM_C_, which indicated that there was no influence of membrane coating on photothermal property (Supplementary Figure S6).

### O_2_ Generation *in vitro*

The *in situ* O_2_ production was measured by using a portable dissolved oxygen meter. As seen from Figure 3G, the DC@ZIF8-MEM and PBS groups did not generate any O_2_, while the DC@ZIF8-MEM_C_ and CAT groups generated time-dependent O_2_ production due to the activity of CAT. Bubbles were visible in the EP tubes of free CAT and DC@ZIF8-MEM_C_, while no bubbles were found with DC@ZIF8-MEM and PBS (Figure 3G). As an oxygen-sensitive dye, the fluorescence of Ru (DPP)_3_Cl_2_ was quenched when O_2_ was present in the solution. With the prolonging of reaction time, the fluorescence intensity of Ru (DPP)_3_Cl_2_ decreased gradually (Figure 3H). When the reaction time was 30 min, the fluorescence intensity of Ru (DPP)_3_Cl_2_ decreased to the lowest.

### Catalytic Activity

CAT is an enzyme that catalyzes the decomposition of H_2_O_2_ to generate O_2_. At 2 h, 68% of H_2_O_2_ was consumed with 2.5 μg·mL^−1^ of free CAT, 64% for DC@ZIF8-MEM_C_ with the same CAT concentration, indicating the same catalytic efficiency as that of free CAT (Figure 3I, Supplementary Figure S7). Comparatively, DC@ZIF8-MEM consumed barely H_2_O_2._ Thus, the catalytic activity of DC@ZIF8-MEM_C_ indeed was derived from CAT. CAT is unstable under physiological conditions, due to enzymic digestion by protein K. At 12 h, the catalytic activity decreased to 34% for free CAT, while 62% was maintained for DC@ZIF8-MEM_C_ (Figure 3J). DC@ZIF8-MEM_C_ is capable of protecting CAT from degradation, which is useful for CAT delivery to maintain catalytic activity *in vivo*.

### Cellular Uptake

**FIGURE 4:**
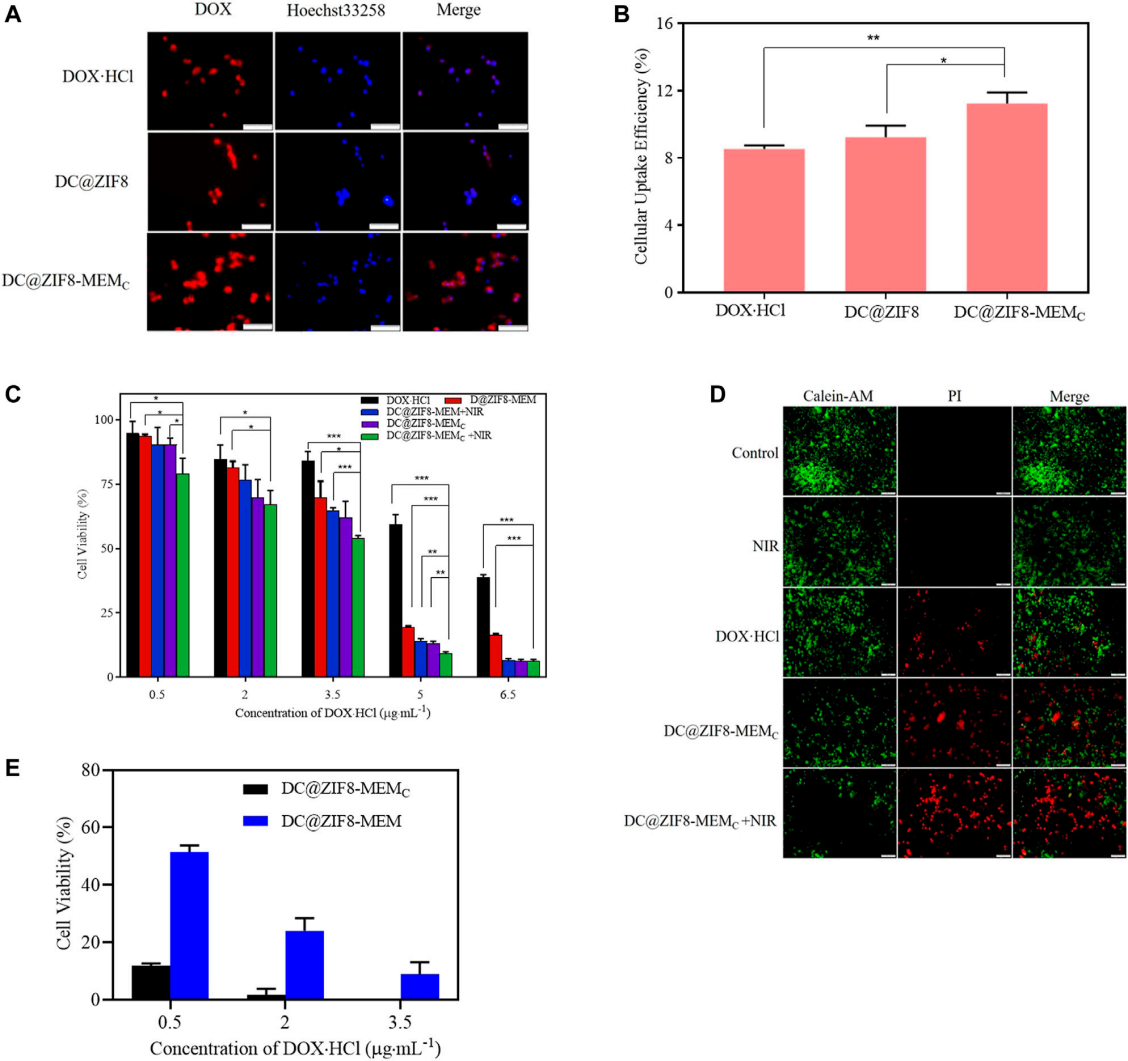
Fluorescence images of mouse breast cancer cells (4T1) incubated with different samples for 4 h, scale bar = 100 µm **(A)**. Cellular uptake efficiency of different samples to 4T1 cells by microplate reader **(B)**. Cytotoxicity of 4T1 cells incubated with different samples at various concentrations for 24 h **(C)**. Labeling of live and dead cells by calcein AM/propidium iodide (PI) for different samples; scale bar = 200 μm **(D)**. Cytotoxicity of 4T1 cells incubated with different samples at various concentrations in hypoxia incubator for 24 h **(E)**. *p < 0.05, **p < 0.01, ***p < 0.001.

To localize the cells, the cell nuclei were stained blue by Heochst-33258. The traffic of DOX·HCl inside the cells could be visualized by its intrinsic red fluorescence (Figure 4A). The fluorescence intensity of DC@ZIF8-MEM_C_ was stronger than that of other treatment groups, presenting the improved cellular uptake through endocytosis (Figure 4A). The relative fluorescence intensities were quantified, which suggested a higher uptake efficiency of DC@ZIF8-MEM_C_, consistent with the result of qualitative analysis (Figure 4B, Supplementary Figure S8). It was attributed from MEM coating, which was helpful for accumulation at the tumor sites by passive targeting (Jiang et al., 2017; Ye et al., 2019).

### Antitumor Activity *in vitro*

The *in vitro* tumor ablation was measured by MTT assays. The cell killing efficiency was dose dependent (Figure 4C). When concentration was at 5 μg·mL^−1^, the cell viability was 59%, 19% for free DOX·HCl, DC@ZIF8-MEM_C_, respectively. Under laser irradiation, the cell viability of groups treated with DC@ZIF8-MEM_C_ decreased to 11%. DC@ZIF8-MEM_C_ indicated that a combination of CT and PTT as well as CAT was better than monotherapy alone.

As seen in Figure 4E, both DC@ZIF8-MEM_C_ and DC@ZIF8-MEM exhibited dose-dependent cytotoxicity. Under hypoxia condition (1% O_2_), compared with DC@ZIF8-MEM, the cell viability of DC@ZIF8-MEM_C_ decreased significantly, demonstrating that the O_2_ produced by CAT could alleviate hypoxia-induced resistance to DOX (12 versus 51% at 0.5 μg·mL^−1^). These results could explain, to some extent, why the treatment effect of DC@ZIF8-MEM_C_ was much better than DC@ZIF8-MEM *in vivo* experiment.

In addition, when the concentration of NPs was 120 μg·mL^−1^, the cell survival rate of C@ZIF8-MEM was 80.0%, and the cell survival rate decreased to 35.0% after NIR (Figure 7A). The results showed photo-responsive cytotoxicity of NPs.

To further evaluate the therapeutic outcome of the NPs, the treated cells were co-stained by calcein-AM and propidium iodide (PI) for live (green) and dead/late apoptotic cells (red), respectively (Liu et al., 2019). Both the control group and NIR group displayed strong green fluorescence (Figure 4D). However, compared with DOX·HCl and DC@ZIF8-MEM_C_ groups, the red fluorescence was predominant for the DC@ZIF8-MEM_C_ + NIR group, displaying a significant number of dead cells, which was in line with the results of cytotoxicity *in vitro* (Figure 4D).

### Intracellular Location

**FIGURE 5:**
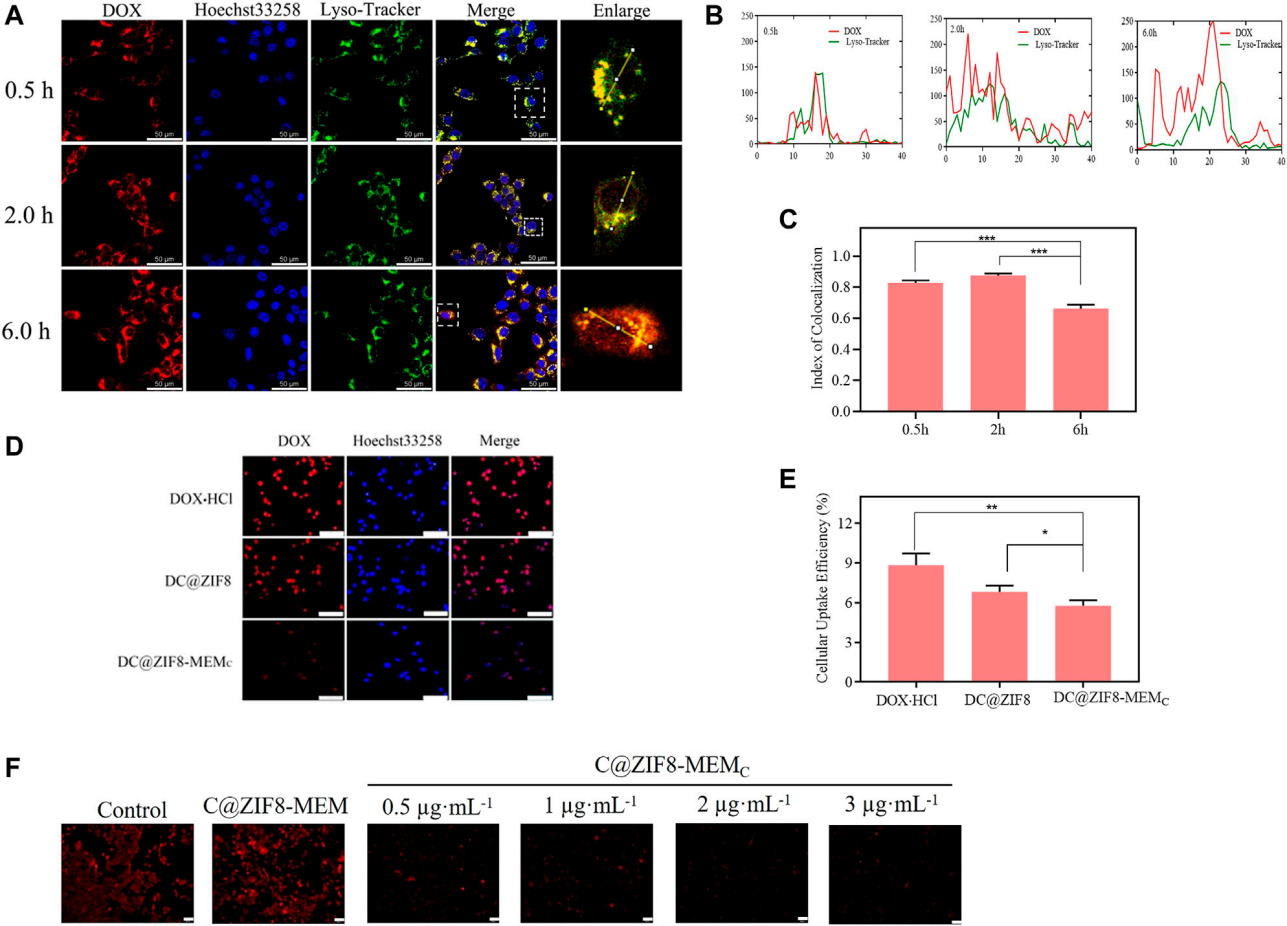
Intracellular distribution fluorescence imaging of DC@ZIF8-MEM_C_ at different times by confocal laser microscopy (CLMS); scale bar = 50 μm **(A)**. Line-scan profiles of DC@ZIF8-MEM_C_ and Lysotracker after 0.5, 2, and 6 h incubation by ImageJ software. The level of the overlapped green line and red line is negatively correlated to endosomal escape of DC@ZIF8-MEM_C_ **(B)**. The index of colocalization of endocytosed DC@ZIF8-MEM_C_ (red) and endosome (green) was calculated by ImageJ software **(C)**. Cellular uptake of different samples incubated with RAW 264.7 cells for 24 h by fluorescent microscope; scale bar = 100 µm **(D)**. Cellular uptake efficiency of different samples to RAW 264.7cells by microplate reader **(E)**. Fluorescence images of 4T1 cells incubated with different samples and then stained with Ru (DPP)_3_Cl_2_; scale bar = 50 μm **(F)**. *p < 0.05, **p < 0.01, ***p < 0.001.

As shown in Figure 5A, there were orange spots that emerged from the red and green channels, an evidence that red fluorescence of the majority of DC@ZIF8-MEM_C_ was in colocalization with green fluorescence (endosomes/lysosomes) at 0.5 h. Over time, the amount of orange spots was weakened, and red color was evenly distributed throughout the cytoplasm, which showed minimal colocalization with the green fluorescence of the endo/lysosomes. The decreased colocalization signal of green and red fluorescence was visualized after 2 h due to the ongoing intracellular transport and rapid endosomal escape. Meaningfully, most of the overlapped signals disappeared after 6 h. The results presented that DC@ZIF8-MEM_C_ entered the lysosome and then escaped into the cytoplasm after uptake into the cell. To semiquantitatively evaluate the capacity of endosomal escape for DC@ZIF8-MEM_C_, the index of colocalization and line scanning profiles of fluorescent intensity of the selected 4T1 cells were calculated by ImageJ software, as given in Figures 5B, C.

These results jointly corroborated the efficient endosomal/lysosomal escape behaviors of DC@ZIF8-MEM_C_, which was attributed to the ZIF8 frame. Under the lysosome pH (pH 4.5–5.5), the ZIF8 moiety could release the 2-methylimidazole ligand, which initiated the “proton-sponge” effect due to the protonation of imidazole rings and resulted in the rupture of lysosome membrane. (Liu Z et al., 2021). The rapid lysosomal escape capability of DC@ZIF8-MEM_C_ was beneficial to maintain the enzymatic activity of CAT and the therapeutic activity of DOX from lysosome to cytoplasm (Varkouhi et al., 2011).

### Immune Evasion

Red fluorescence was strong for DOX·HCl group, while weak it was for DC@ZIF8-MEM_C_ group (Figure 5D). As shown in Figure 5E, the uptake rate of DC@ZIF8-MEM_C_ by RAW 264.7 cells were significantly lower than those of the other two groups, displaying that NPs camouflaged by blood cell membranes could avoid the phagocytosis of immune cells. As natural stealth coating, the cell membrane could alleviate immunogenicity and enhance biocompatibility (Graham and Unger, 2018).

### Intracellular Oxygen Determination

Oxygen probe Ru(DPP)_3_Cl_2_ was applied for intracellular determination. The fluorescence of Ru(DPP)_3_Cl_2_ would be weakened in the presence of abundant O_2_. As from Figure 5F, compared with the control group, the red fluorescence of C@ZIF8-MEM was strong and predominant, while that of the C@ZIF8-MEM_C_ group was weak and marginal. For C@ZIF8-MEM without embedded CAT, the fluorescence of Ru (DPP)_3_Cl_2_ barely changed. However, the fluorescence change was weak due to the O_2_ generation for the C@ZIF8-MEM_C_ group. Moreover, the fluorescence tended to be weaker with the increase in CAT concentration of C@ZIF8-MEM_C._ The results verified that the intracellular O_2_ generation of C@ZIF8-MEM_C_ originated from embedding the CAT.

### Antitumor Effect *in vivo*

**FIGURE 6:**
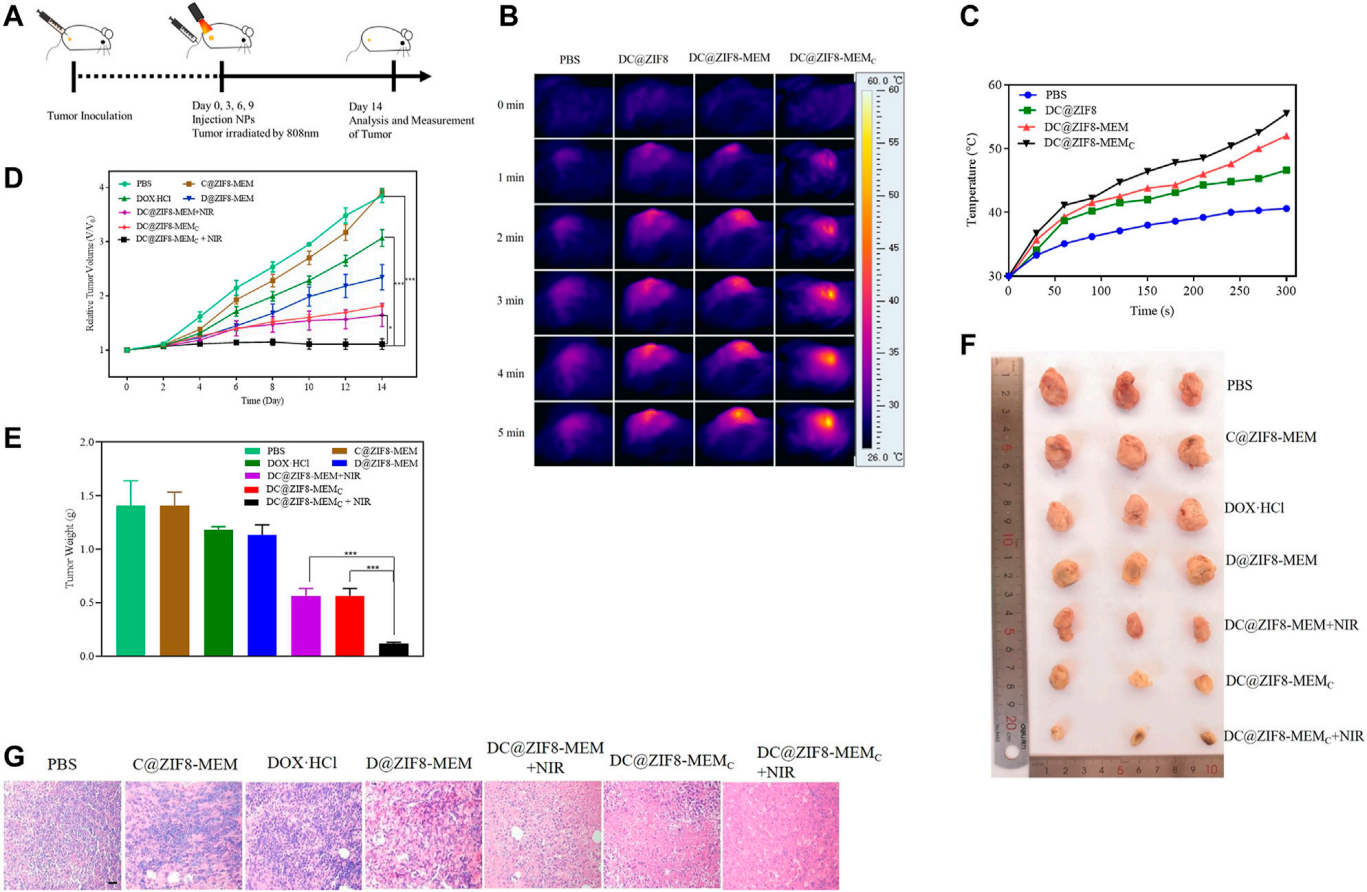
Experimentation for the therapeutic model **(A)**. Photothermal images *in vivo* **(B)**. Temperature curves with 808 nm NIR at 2 W·cm^−2^ for 5 min **(C)**. Relative tumor volumes of mice with different treatments **(D)**. Tumor weights of mice after different treatments **(E)**. Representative digital photographs of excised tumor tissues of mice with different treatments **(F)**. H&E-stained images of tumor tissues from mice after different treatments on the 14th day; scale bar = 20 µm **(G)**. All the above data on mice in each group were averagely calculated (n = 5). *p < 0.05, **p < 0.01, ***p < 0.001.

The experimentation for the therapeutic model is shown in Figure 6A. The antitumor effect was monitored by measuring the tumor size using a caliper every other day. With PBS or C@ZIF8-MEM treatment, the tumor grew rapidly over time (Figure 6D). Without irradiation, the DC@ZIF8-MEM_C_ showed moderate tumor inhibition due to the CT of DOX·HCl and hypoxic improvement of CAT. Combined with irradiation, the tumor inhibition effect was significantly elevated (Figure 6D). The tumor weight was only 0.12 g for the DC@ZIF8-MEM_C_ + NIR group, while the tumor weights were 1.19, 1.13, 0.57, and 0.57 g for DOX·HCl, DC@ZIF8-MEM, DC@ZIF8-MEM + NIR, and DC@ZIF8-MEM_C_, respectively (Figure 6E). It could be clearly seen from the photographs of the extracted tumor tissues after treatment that the tumors treated with DC@ZIF8-MEM_C_ plus irradiation were ablated significantly (Figure 6F). Interestingly, compared with the DC@ZIF8-MEM + NIR, the DC@ZIF8-MEM_C_ + NIR group exhibited higher tumor inhibition effect, which may be attributed to amelioration of CAT for tumor hypoxia (Figures 6D–F). The results were consistent with cytotoxicity *in vitro*. Upon irradiation, DC@ZIF8-MEM_C_ displayed the most effectivity in tumor inhibition, superior to other groups, indicating the advantages of combined cancer therapy. Tumor slices had a large area of necrosis for the DC@ZIF8-MEM_C_ + NIR group, consistent with the therapeutic effect (Figure 6G).

### Photothermal Effect *in vivo*

After continuous irradiation of the tumor site with the 808 nm near-infrared laser (2 W·cm^−2^) for 5 min, the near-infrared imaging image of the tumor site is shown in Figure 6B. The infrared light intensity of the tumor site for the DC@ZIF8-MEM_C_ group was significantly stronger than that of the other groups. Figure 6C shows the temperature rise curves of each group. The temperature of the DC@ZIF8-MEM group and the DC@ZIF8-MEM_C_ group changed rapidly within 1 min. After 5 min, the temperature of the tumor site increased to 40.1°C, 46.6°C, 52°C, and 55.5°C for PBS, DC@ZIF8, DC@ZIF8-MEM, and DC@ZIF8-MEM_C_, respectively (Figure 6C). It presented that DC@ZIF8-MEM_C_ played an effective photothermal effect.

### Biocompatibility and Safety

**FIGURE 7:**
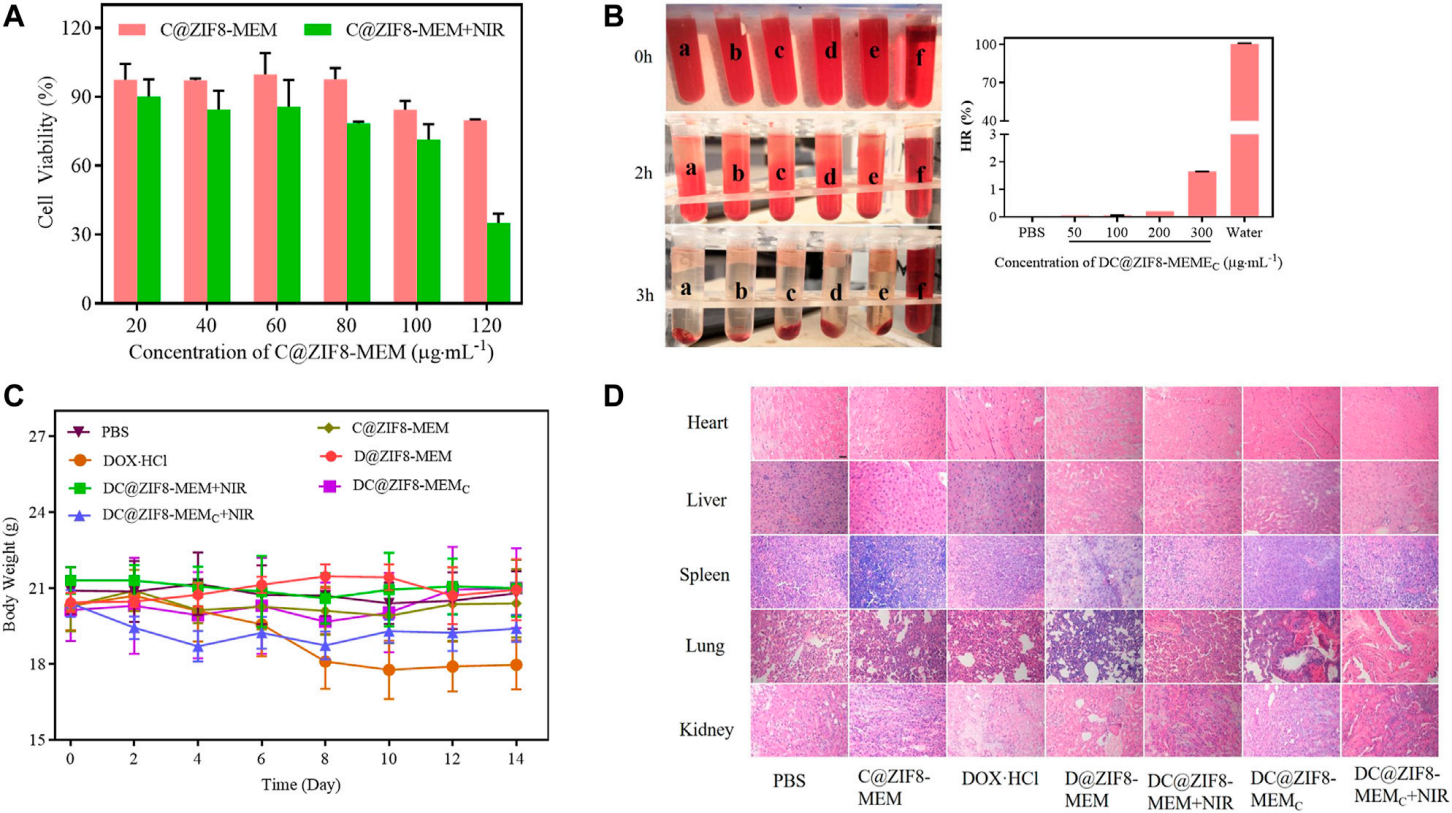
Cell viability of C@ZIF8-MEM to 4T1 cells with and without NIR **(A)**. The hemolytic image (left) and hemolysis ratio of samples (right), PBS (a), 50 μg·mL^−1^ DC@ZIF8-MEM_C_ (b), 100 μg·mL^−1^ DC@ZIF8-MEM_C_ (c), 200 μg·mL^−1^ DC@ZIF8-MEM_C_ (d), 300 μg·mL^−1^ DC@ZIF8-MEM_C_ (e), deionized water (f) **(B)**. Body weights of mice with different treatments **(C)**. H&E analysis of major organs from mice after treatments; scale bar = 20 µm **(D)**.

The biocompatibility of the NPs was evaluated. The cell viability of C@ZIF8-MEM displayed more than 80%, exhibiting good biocompatibility (Figure 7A, Supplementary Figure S9). The positive control (deionized water) showed obvious hemolysis, with HR as high as 100%. There was no hemolysis found for DC@ZIF8-MEM_C_ and PBS. HR of DC@ZIF8-MEM_C_ was less than 2% at a concentration as high as 300 μg·mL^−1^ (Figure 7B). Therefore, DC@ZIF8-MEM_C_ was highly biocompatible and could be directly administered by intravenous injection. There was no obvious decrease in body weight for all treatment groups except the DOX·HCl group (Figure 7C). Additionally, no significant histopathology changes were noticed for the main organs such as the heart, liver, spleen, lungs, and kidneys, suggesting invisible short-term toxicity during treatments (Figure 7D). These results demonstrated DC@ZIF8-MEM_C_ as promising for biomedicine application with high bio-safety.

## Conclusion

In summary, biomimic DC@ZIF8-MEM_C_ was established and investigated. High DOX·HCl loading and self-generation of O_2_ which enhanced CT and PTT, were realized. Furthermore, the superior controlled drug release, photothermal efficiency, and excellent biocompatibility of DC@ZIF8-MEM_C_ were retained for remarkable antitumor effect of chemo-thermo synergistic therapy. Our study sheds light on the great chemo-photothermal synergistic antitumor effect under the sensitization of CAT.

## Data Availability

The original contributions presented in the study are included in the article/Supplementary Material, further inquiries can be directed to the corresponding authors.
