# Supplementary material for: Biomimetic ZIF8 Nanosystem With Tumor Hypoxia Relief Ability to Enhance Chemo-Photothermal Synergistic Therapy
Source: Front Pharmacol. 2022 Mar 24;13:850534. doi: 10.3389/fphar.2022.850534 (PMC8988193; doi:10.3389/fphar.2022.850534)
Supplement: Supplementary file 1 [file DataSheet1.PDF]

Supply Information

Biomimetic ZIF-8 Nanosystem with Tumor Hypoxia Relief Ability to Enhance Chemo-photothermal Synergistic Therapy

Ziming Zhao[1,2] Zhaorong Liu[1] Yabing Hua[1,2] Yuanjie Pan[1] Ge Yi[1] Shengyue Wu[1]

Cong He[1] Yanzhuo Zhang[1,2*] Yihua Yang[1,2*]

[1] Jiangsu Key Laboratory of New Drug Research and Clinical Pharmacy, Xuzhou Medical University, Xuzhou 221004, China

[2] Department of Pharmaceutics, School of Pharmacy, Xuzhou Medical University, Xuzhou 221004, China

*Corresponding authors.

Email addresses: yanzhuozhang@126.com (YZ. Zhang), nancyyyh@126.com (YH. Yang).

1. Appearance of CuS NPs, DC@ZIF8 and DC@ZIF8-MEM_C

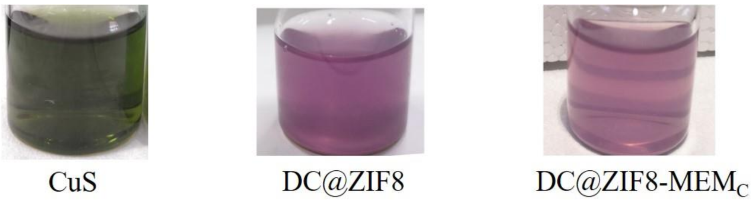

Figure S1. Appearance of CuS NPs, DC@ZIF8 and DC@ZIF8-MEM_C.

2. Morphology of CuS NPs and DC@ZIF8

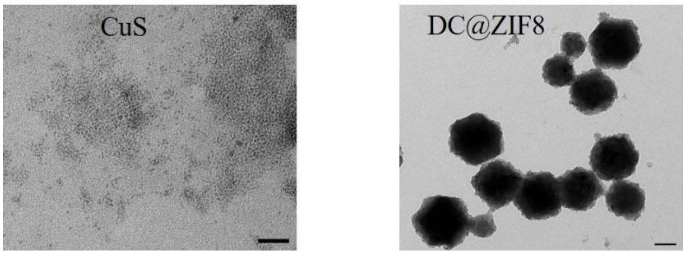

Figure S2. Representative TEM images of CuS NPs (scale bar=50nm) and DC@ZIF8 (scale bar=100nm).

3. Determination of DOX·HCl by fluorescence spectrophotometer

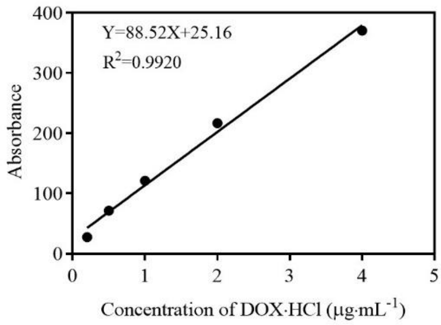

Figure S3. Linear relationship between the fluorescence intensity and the DOX·HCl concentration by fluorescence spectrophotometer.

4. Determination of CAT by ammonium molybdate colorimetric method

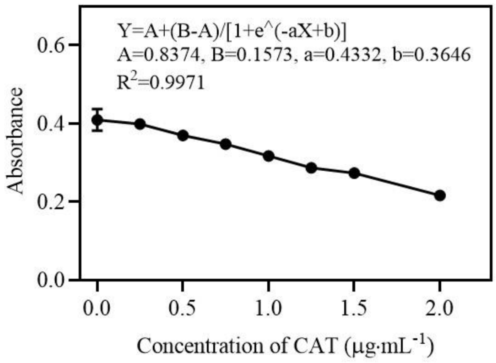

Figure S4. Linear relationship between absorbance and the CAT concentration by ammonium molybdate colorimetric method.

5. Colloidal stability of DC@ZIF8-MEM_C

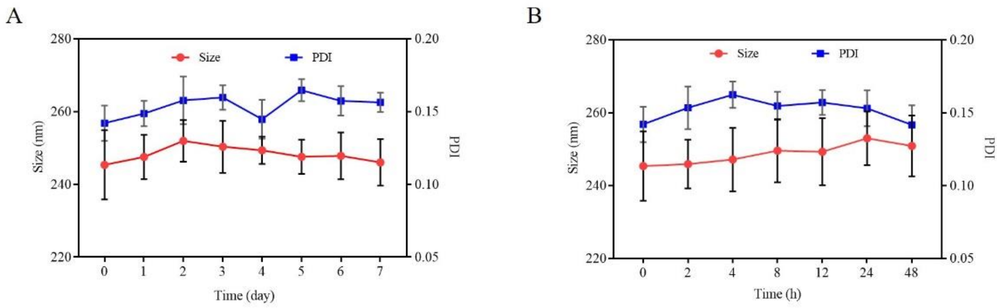

Figure S5. Colloidal stability of DC@ZIF8-MEM_C. Size and PDI of DC@ZIF8-MEM_C after incubation with PBS for 7 days at 4℃ (A) and incubation with 10% FBS for 48 h at 37 ℃ (B).

6. Photothermal activity of DC@ZIF8 *in vitro*

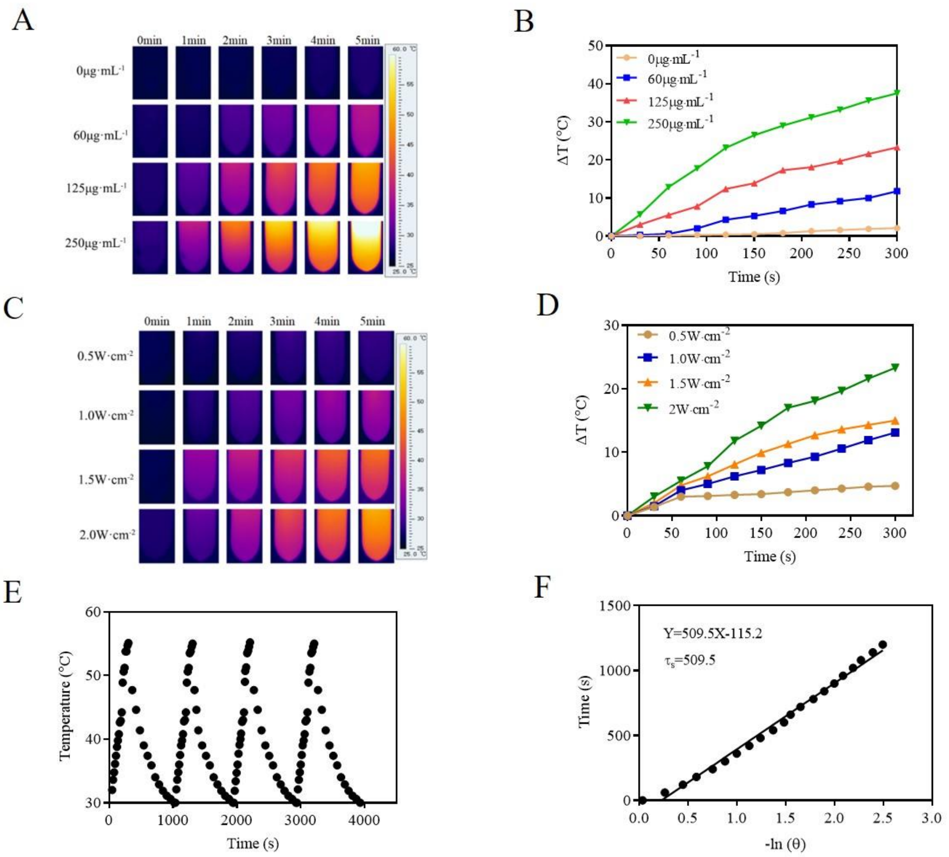

Figure S6. Photothermal images of DC@ZIF8 with various concentration by FLIR (A); Temperature curves by changing concentration of DC@ZIF8 with 808nm NIR at $2W·cm^{-2}$ for 5 min(B); Photothermal images of DC@ZIF8 with various laser power density by FLIR (C); Temperature curves of DC@ZIF8 by changing the laser power density (D); Temperature variations of DC@ZIF8 by repeating the on/off ($2W·cm^{-2}$) (E); Temperature fitting curve of DC@ZIF8 (F).

7. H_2O_2 consumption with different concentration of free CAT

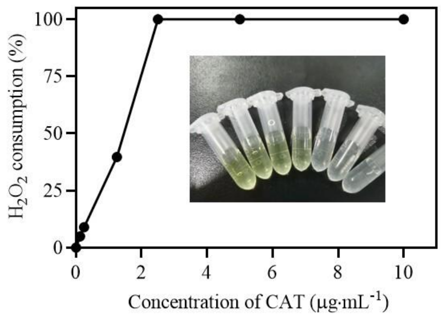

Figure S7. H_2O_2 consumption after it mixing with series concentrations of free CAT. H_2O_2 residue was determined by adding ammonium molybdate. Inset: Photos of the color of the solutions after adding with ammonium molybdate.

8. Determination of DOX·HCl by microplate reader

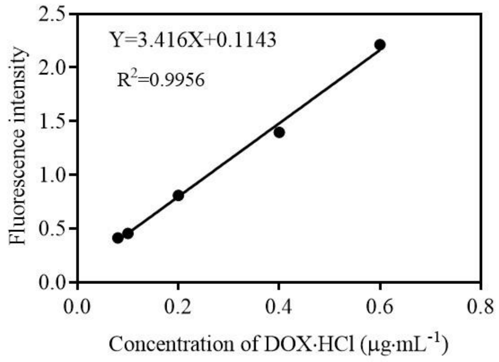

Figure S8. Linear relationship between the fluorescence intensity and the DOX·HCl concentration by microplate reader.

9. Biocompatibility of C@ZIF8-MEM

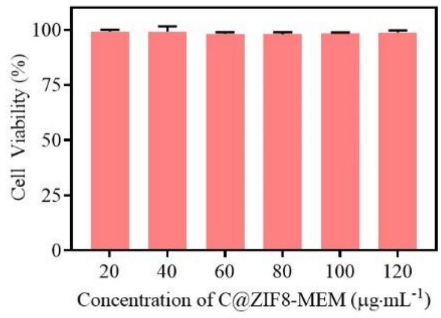

Figure S9. Cell viability of C@ZIF8-MEM after incubation with LO2 cells for 24 h.
